# Prediction of pathological complete response to neoadjuvant chemotherapy in patients with breast cancer using a combination of contrast‐enhanced ultrasound and dynamic contrast‐enhanced magnetic resonance imaging

**DOI:** 10.1002/cam4.5019

**Published:** 2022-07-13

**Authors:** Xue Han, Huajing Yang, Shiyang Jin, Yunfeng Sun, Hongxia Zhang, Ming Shan, Wen Cheng

**Affiliations:** ^1^ Department of Ultrasound Harbin Medical University Cancer Hospital Harbin China; ^2^ Department of Breast Surgery Harbin Medical University Cancer Hospital Harbin China; ^3^ Imaging Center Harbin Medical University Cancer Hospital Harbin China

**Keywords:** contrast‐enhanced, dynamic contrast‐enhanced MRI, neoadjuvant chemotherapy, ultrasound, ultrasound breast cancer

## Abstract

This study aimed to evaluate the value of dynamic contrast‐enhanced ultrasound (CEUS) combined with dynamic contrast‐enhanced magnetic resonance imaging (DCE‐MRI) in predicting pathological complete response (pCR) in patients with breast cancer receiving neoadjuvant chemotherapy (NAC). Fifty‐seven female patients with breast cancer (mean age, 50.46 years; range, 32–66 years) scheduled for NAC were recruited. CEUS and DCE‐MRI were performed before and after NAC. Imaging features and their changes were compared with postoperative pathological results. After the clinical differences were balanced using propensity score matching, univariate and multiple logistic regression analyses were used to derive the characteristics independently associated with pCR. Receiver operating characteristic curve analysis was performed to assess diagnostic performance. After six to eight cycles of NAC, 24 (42.1%) patients achieved pCR, while 33 (57.9%) did not. Multivariate analysis showed that enhancement order on CEUS and DCE‐MRI before NAC, reduction in diameter and enhancement shape on CEUS, maximum diameter on DCE‐MRI, and the type of progressive dynamic contrast enhancement after NAC were independently associated with pCR after NAC. The area under the receiver operating characteristic curve for CEUS+DCE‐MRI was 0.911 (95% confidence interval, 0.826–0.997), and the specificity and positive predictive values were 87.0% and 87.5%. CEUS and DCE‐MRI have the potential for assessing the pathological response to NAC in patients with breast cancer; their combination showed the best diagnostic performance. CEUS+DCE‐MRI has proved beneficial for comprehensive assessment and personalizing treatment strategies for patients with breast cancer.

## Graphical abstract

Our study evaluated the ability of contrast‐enhanced ultrasound and dynamic contrast‐enhanced magnetic resonance imaging to predict pathological complete response to neoadjuvant chemotherapy in patients with breast cancer.

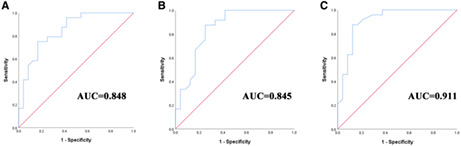

## 1 INTRODUCTION

Breast cancer is the second leading cause of female death and is a major threat to the health of women.^1^ Approximately half of a million women die of breast cancer each year, and its incidence is increasing.^2^ Surgery, radiation therapy, and chemotherapy are the main breast cancer treatments.^3^ Currently, the development of accurate diagnostic methods and the prediction of treatment outcomes have become hot topics in breast cancer research.^4^

Currently, neoadjuvant chemotherapy (NAC) has been widely used as an important component of systemic treatment before surgery or radiotherapy for locally advanced breast cancer.^5^ NAC increases the success likelihood of breast‐conserving surgery, and its response can be used to predict patient outcomes after surgery.^6^ Pathological complete response (pCR) is considered an indicator of higher overall survival and long‐term disease‐free survival in patients receiving NAC.^7^ Thus, an effective and accurate method for pCR assessment is urgently required.

Dynamic contrast‐enhanced magnetic resonance imaging (DCE‐MRI) describes tumor shape and functional information and can be used to visualize altered angiogenesis, which is closely related to tumor progression.^8^ The pathophysiological mechanisms used for response assessment by DCE‐MRI are associated with changes in microvessel density and the anti‐angiogenic effect of chemotherapy.^9^ Several reports have demonstrated an association between NAC response and DCE‐MRI features.^10^ While DCE‐MRI is an imaging modality with high diagnostic performance, it is time‐consuming and expensive.

As a relatively low‐cost imaging method, dynamic contrast‐enhanced ultrasound (CEUS) has attracted extensive attention for assessing microvascular morphology and distribution features of breast tumors and overcoming the limitations of conventional ultrasonography.^11^ Unlike the gadolinium‐based extracellular contrast used for MRI, the CEUS contrast agent remains within the blood vessels. Given the angiogenesis that occurs in capillaries, CEUS may be more accurate for visualizing changes in tumor perfusion.^12^ The use of CEUS to estimate tumor response to NAC in patients with breast cancer has been previously reported.^13^, ^14^ However, this study is among the few to compare the diagnostic performances of CEUS, DCE‐MRI, and their combination.

Our present study aimed to accurately predict pCR among patients with breast cancer using combined CEUS and DCE‐MRI.

## 2 METHODS

### 2.1 Patients

**FIGURE 1:**
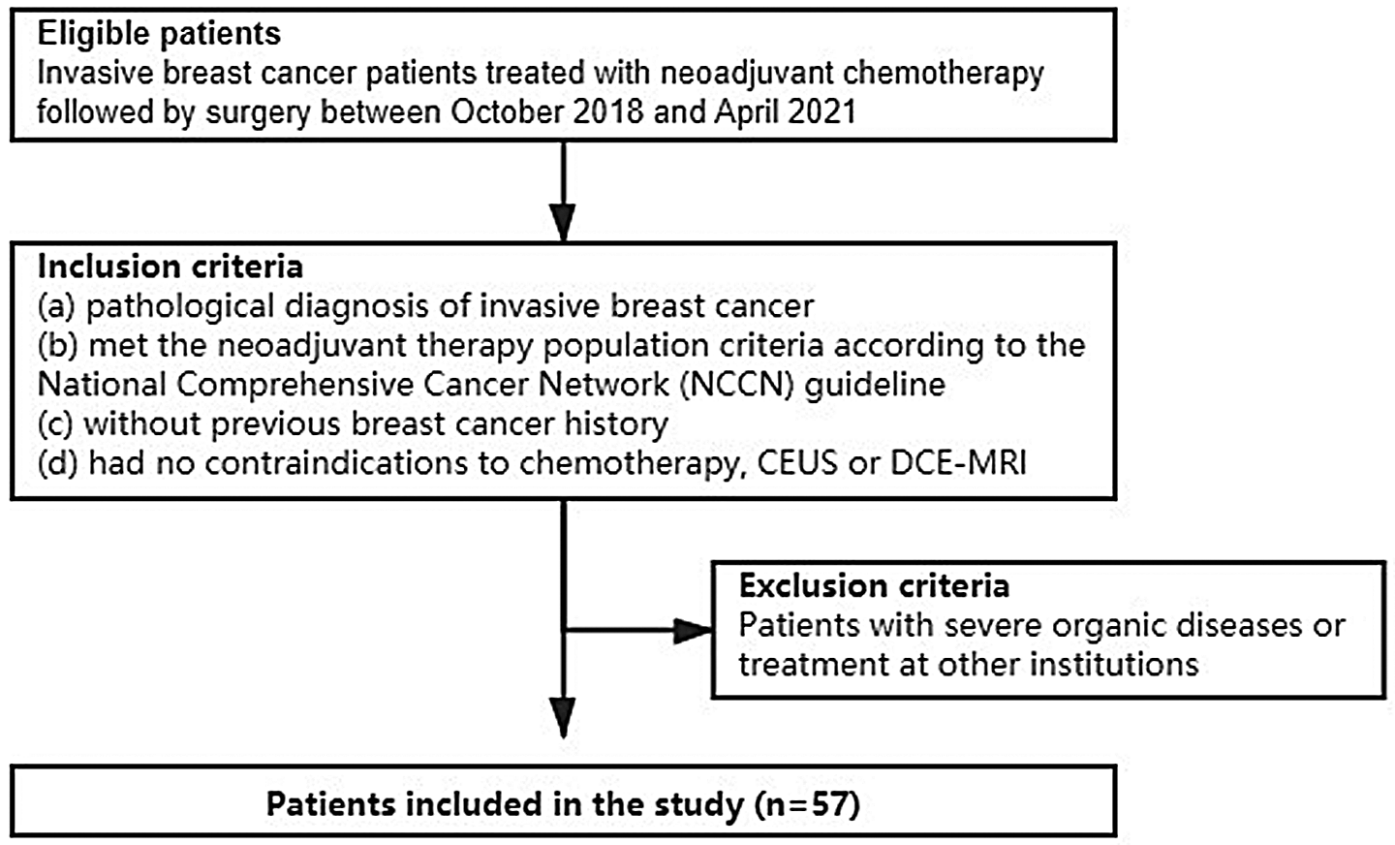
Flowchart for the inclusion and exclusion criteria. CEUS, contrast‐enhanced ultrasonography; DCE‐MRI, dynamic contrast‐enhanced magnetic resonance imaging; NAC, neoadjuvant chemotherapy.

This retrospective study was approved by the ethics committee of Harbin Medical University Cancer Hospital, and informed consent was obtained from all patients. From October 2018 to April 2021, 57 female patients (mean age, 50.46 years; range, 32–66 years) with biopsy‐proven invasive breast cancer were recruited for our study, and they underwent examinations of CEUS and DCE‐MRI before and after six to eight cycles of NAC. The inclusion criteria included the following conditions: (a) pathological diagnosis of invasive breast cancer; (b) meeting the neoadjuvant therapy population criteria according to the National Comprehensive Cancer Network guidelines; (c) no previous breast cancer history; and (d) no contraindications for chemotherapy, CEUS, or DCE‐MRI. Patients with severe organic diseases or those treated at other institutions were excluded from the present study (Figure 1).

All patients underwent anthracycline‐ and taxane‐based NAC, and patients (*n* = 18) with HER2‐positive cancers received Herceptin, a targeted drug. Subsequently, breast‐conserving surgery or mastectomy was performed, and axillary lymph nodes were removed by sentinel lymph node biopsy or axillary lymph node dissection.

### 2.2 CEUS data acquisition and analysis

CEUS was performed using Canon Aplio i900 (Canon Medical Systems USA, Inc.) and an i18LX5 ultrasound linear transducer (Canon Medical Systems USA, Inc.) with a frequency of 5.5 MHz, and the contrast agent used in the study was SonoVue (Bracco). After 4 ml of SonoVue suspension was injected intravenously from the patient's anterior elbow, 5 ml of saline was introduced to flush the catheter. The dynamic image was stored after injection of the contrast agent, and contrast scanning was continued until the end of lesion enhancement.

Two highly experienced doctors (with more than 10 years' experience in CEUS) retrospectively analyzed the CEUS images without knowing the pathological results. Two doctors made conclusions after independent explanations and reaching a consensus if there was disagreement.

### 2.3 DCE‐MRI data acquisition and analysis

MRI examinations were performed using a 3.0‐T magnetic resonance imaging system (Philips Ingenia) with a 7‐channel breast coil in our study. Gadobutrol (Bayer) was intravenously injected (1.0 ml/s) at a dose of 0.2 mmol/kg of body weight, and 20 ml of saline solution was used to flush the catheter. Axial T1‐weighted fat‐suppression images (TR, 36 ms, TE 4.6 ms, slice thickness 1.0 mm, matrix, 250 × 350 × 180; field of view, 250 mm) were obtained. Dynamic contrast‐enhanced MR examination was performed 90 s after injection of the contrast agent. The scan time was 90 s/scan, and the total duration was 9 min.

The character analysis of DCE‐MRI performance was also performed by two radiologists (with 10 and 6 years of experience, respectively), and they were blinded to the patient's pathological results. According to previous studies,^15^ the enhancement patterns and parameters of CEUS and DCE‐MRI were classified as follows: (1) Internal homogeneity: A, homogeneous enhancement: total and diffuse enhancement of the tumor; B, heterogeneous enhancement: uneven enhancement of the tumor; (2) Enhancement degree: A, hyper‐enhancement: tumor lesion was more enhanced than normal breast tissue; B, iso‐enhancement/hypo‐enhancement: tumor lesion had equal or lower enhancement compared to normal breast tissue; (3) Enhancement order: A, central enhancement: enhancement originating from center of the tumor; B, peripheral enhancement: enhancement originating from periphery of the tumor; (4) Enhancement margin: A, clear margin: more than half the margin of tumor lesion was clearly visible; B, blurred margin: less than half the margin of tumor lesion was clearly visible; (5) Enhancement shape: Regular shape: the enhanced lesion was regular oval; B, irregular shape: the enhanced lesion was irregular; (6) Lymphadenopathy at ultrasound: A, positive: images showing swollen lymph nodes with cortex of lymph node ≥3 mm or lymph node aspect ratio <2; B, negative: images showing no abnormally swollen lymph nodes; (7) Lymphadenopathy at MRI: A, positive: images showing swollen lymph nodes with diameter more than 10 mm and deficiency of fat signal; B, negative: images showing no abnormally swollen lymph nodes; (8) DCE curve types: A, progressive enhancement: during the observation time, the signal intensity continued slowly; B, plateau: early rapid and significant enhancement peaked, with no significant change in signal intensity during the delay period (range ± 10%); washout: early rapid and significant enhancement peaked, with signal intensity decreasing rapidly during the delay period (decrease >10%).

### 2.4 Pathological examination

Pathological assessment and assessment of NAC response were performed by a pathologist with more than 15 years of experience. A fine needle biopsy was used to determine pathological and immunohistochemical types. The expressions of prognostic indicators, including estrogen receptor (ER), progesterone receptor (PR), and human epidermal growth factor receptor‐2 (HER2), were identified by immunohistochemical staining.^16^, ^17^ After surgery, pCR was defined as the absence of invasive residual cancer, with or without ductal carcinoma in situ.^18^

### 2.5 Statistical analysis

All data analyses were evaluated by SPSS version 26 (IBM Corp.). Propensity score matching (PSM) was performed using R (Version 4.1.2) to balance the clinical discrepancy between pCR and non‐pCR groups before logistic regression analyses. Univariate analyses of the CEUS and DCE‐MRI features of breast tumors were evaluated using independent‐samples *t*‐test or nonparametric tests for continuous variables. Chi‐squared or Fisher's exact test was performed for analyzing categorical variables. Univariate and multiple logistic regression analysis were performed to identify the features that were independently associated with pCR. Receiver operating characteristic (ROC) curve analysis was performed to assess the diagnostic capabilities and determine the sensitivity, specificity, accuracy, positive predictive value (PPV), and negative predictive value (NPV). The area under the curve (AUC) was obtained, and *p* < 0.1 was considered statistically significant.

## 3 RESULTS

### 3.1 Patient characteristics and pathological responses to NAC

Among the 57 patients, 24 (42.1%) achieved pCR after six to eight cycles of NAC, and 33 (57.9%) did not. The patients' clinical characteristics are shown in Table 1. Regarding the clinical characteristics, the non‐pCR group had more initial clinical stage II (60%, *p* = 0.066), mastectomy type (93.9%, *p* = 0.059), and axillary lymph node dissection (81.8%, *p* = 0.011) than the pCR group. Regarding the pathological characteristics, the pCR group had more ER negativity (79.8%, *p* = 0.010) and triple‐negative cancer subtypes (50.0%, *p* = 0.023) than the non‐pCR group. The two groups did not show a significant difference in ages, PR statuses, HER2 statuses, and Ki‐67 indexes (all *p* ≥ 0.1). After PSM, the clinical discrepancy between pCR and non‐pCR groups was balanced.

**TABLE 1:** Patients and clinical characteristics before and after propensity score matching

Characteristic	Before propensity score matching				After propensity score matching			
	pCR (*n* = 24)	non‐pCR (*n* = 33)	*p* value	SMD	pCR (*n* = 24)	non‐pCR (*n* = 24)	*p* value	SMD
Age	51.88 ± 9.77	49.42 ± 7.86	0.299	0.276				0.226
Clinical stage			0.066	0.661				0.183
I	3 (12.5%)	2 (6.1%)						
II	19 (79.2%)	20 (60.6%)						
III	2 (8.3%)	11 (33.3%)						
Tumor surgery type			0.059	0.542				0.459
Breast‐conserving surgery	6 (25.0%)	2 (6.1%)						
Mastectomy	18 (75.0%)	31 (93.9%)						
Axillary surgery type			0.011	0.713				0.535
SLNB	12 (50.0%)	6 (18.2%)						
ALND	12 (50.0%)	27 (81.8%)						
ER status			0.010	0.737				0.436
Negative	17 (70.8%)	12 (36.4%)						
Positive	7 (29.2%)	21 (63.6%)						
PR status			0.294	0.285				0.087
Negative	15 (62.5%)	16 (48.5%)						
Positive	9 (37.5%)	17 (51.5%)						
HER2 status			0.808	0.065				0.173
Negative	16 (66.7%)	23 (69.7%)						
Positive	8 (33.3%)	10 (30.3%)						
Subtype			0.023	0.796				0.454
Hormone‐positive	4 (16.7%)	16 (48.5%)						
HER2‐positive	8 (33.3%)	10 (30.3%)						
Triple‐negative	12 (50.0%)	7 (21.2%)						
Ki‐67 index (%)	37.3 ± 20.5	37.4 ± 18.5	0.980	0.007				0.123

Abbreviations: ALND, axillary lymph node dissection; SLNB, sentinel lymph node biopsy; SMD, Standardized mean difference. Data are expressed as the mean ± standard deviation or *n* (%), and *p* < 0.1 was considered statistically significant.

### 3.2 Univariate and multivariate analysis

**FIGURE 2:**
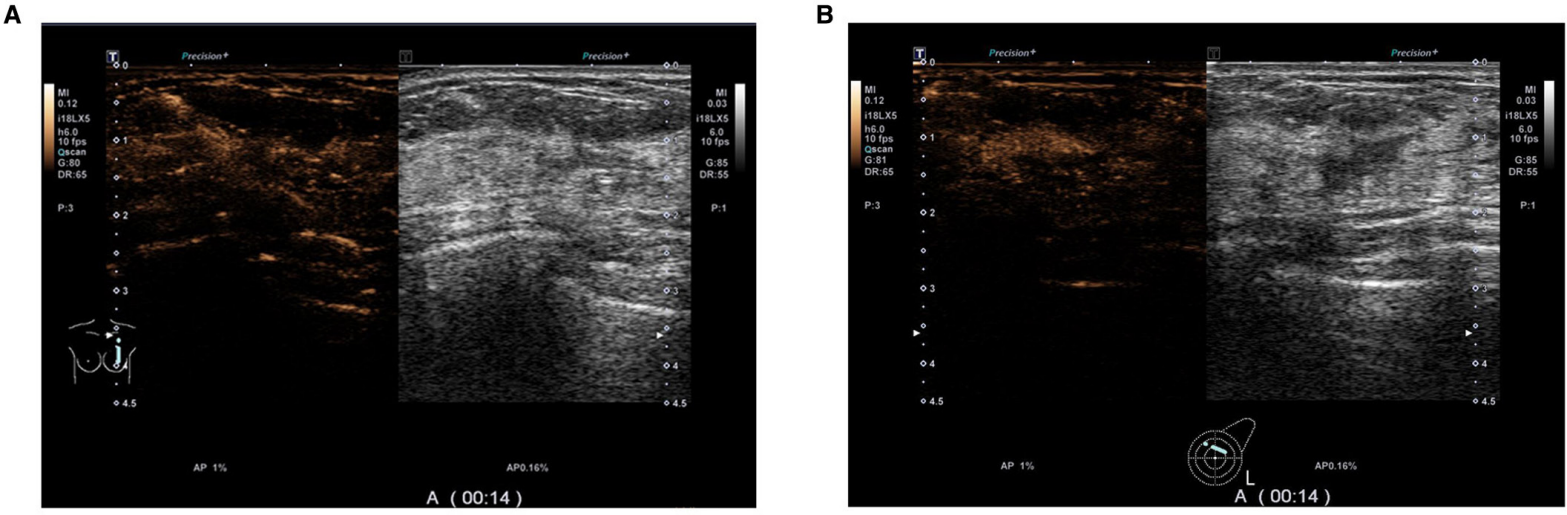
CEUS images of a 46‐year‐old patient with breast cancer with pathological result of pCR. The image before NAC shows heterogeneous hyper‐enhancement of the breast tumor (A), while that after NAC shows homogeneous hypo‐enhancement of the breast tumor (B). The maximum tumor diameter decreased from 38 mm to 10 mm, a tumor size change of 74%.

**FIGURE 3:**
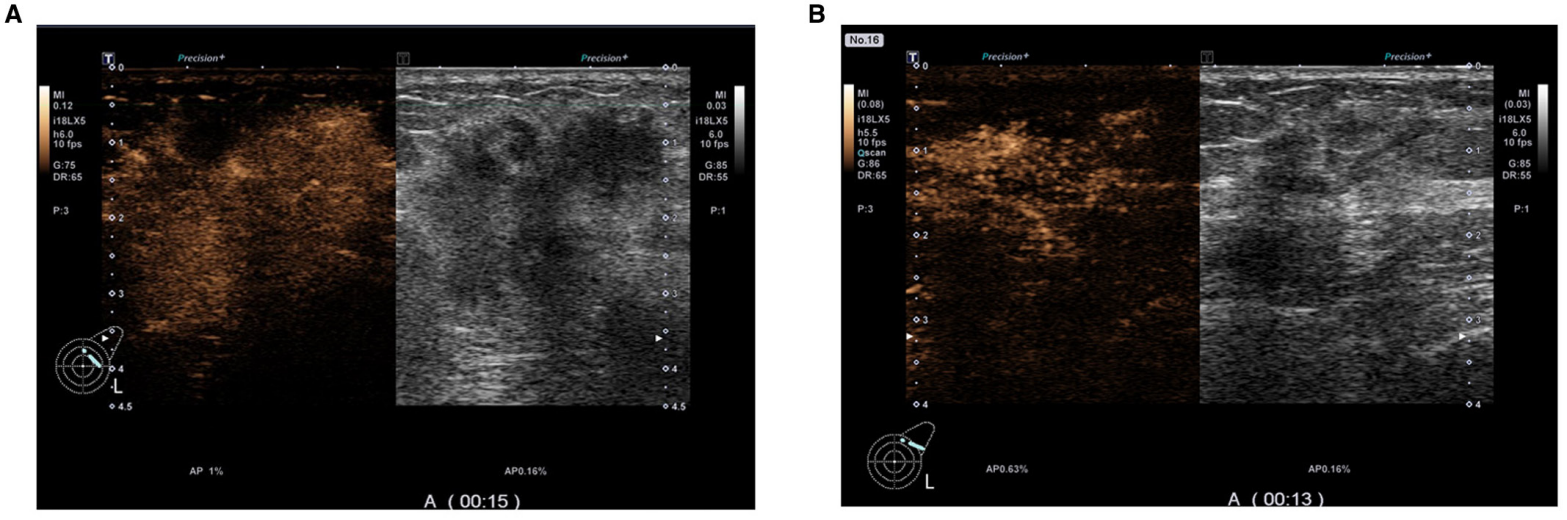
CEUS images of a 62‐year‐old patient with breast cancer with pathological result of non‐pCR. Images before NAC (A) and after NAC (B) both show heterogeneous hyper‐enhancement of the breast tumor with blurred margins and irregular shape. The maximum tumor diameter decreased from 40 mm to 28 mm, a tumor size change of 30%.

The univariate analysis of CEUS features (Table 2) showed that central enhancement of breast tumors before and after NAC was associated with non‐pCR (both, *p* < 0.1). In addition, the pCR group showed higher proportions of lower maximum diameter (10.2 mm vs. 12.8 mm, *p* = 0.017), reduction in diameter (63.8% vs. 56.5%, *p* = 0.022), homogeneous enhancement (9 vs. 1, *p* = 0.010), iso‐enhancement/hypo‐enhancement (22 vs. 15, *p* = 0.036), clear margin (10 vs. 2, *p* = 0.080), and regular shape (11 vs. 2, *p* = 0.008) of enhanced tumor after NAC (Figures 2 and 3).

**TABLE 2:** CEUS features before and after NAC according to pathological response

CEUS features	Before NAC			After NAC		
	pCR (*n* = 24)	non‐pCR (*n* = 24)	*p* value	pCR (*n* = 24)	non‐pCR (*n* = 24)	*p* value
Maximum diameter (mm)	30.8 ± 11.4	31.8 ± 12.7	0.587	10.2 ± 4.4	12.8 ± 7.5	0.017
Reduction in diameter (%)	–	–	–	63.8 ± 17.9	56.5 ± 21.9	0.022
Internal homogeneity			1.000			0.010
Homogeneous enhancement	2(8.3%)	1(4.2%)		9(37.5%)	1(4.2%)	
Heterogeneous enhancement	22(91.7%)	23(95.8%)		15(62.5%)	23(95.8%)	
Enhancement degree			0.771			0.036
Hyper‐enhancement	10(41.7%)	11(45.8%)		2(8.3%)	9(37.5%)	
Iso‐enhancement/hypo‐enhancement	14(58.3%)	13(54.2%)		22(91.7%)	15(62.5%)	
Enhancement order			0.042			0.042
Central enhancement	10(41.7%)	17(70.8%)		7(29.2%)	14(58.3%)	
Peripheral enhancement	14(58.3%)	7(29.2%)		17(70.8%)	10(41.7%)	
Enhancement margin			1.000			0.080
Clear margin	1(4.2%)	2(8.3%)		10(41.7%)	2(8.3%)	
Blurred margin	23(95.8%)	22(91.7%)		14(58.3%)	22(91.7%)	
Enhancement shape			1.000			0.008
Regular shape	1(4.2%)	1(4.2%)		11(45.8%)	2(8.3%)	
Irregular shape	23(95.8%)	23(95.8%)		13(54.2%)	22(91.7%)	
Lymphadenopathy			0.724			0.318
Positive	18(75.0%)	20(83.3%)		16(66.7%)	20(83.3%)	
Negative	6(25.0%)	4(16.7%)		8(33.3%)	4(16.7%)	

Data are expressed as the mean ± standard deviation or *n* (%), and *p* < 0.1 was considered statistically significant.

The univariate analysis of DCE‐MRI features (Table 3) showed that central enhancement of breast tumors before NAC was associated with non‐pCR (*p* = 0.042). After NAC, a lower maximum diameter (11.3 mm vs. 15.0 mm, *p* = 0.005), reduction in diameter (58.7% vs. 51.1%, *p* = 0.013), iso‐enhancement/hypo‐enhancement (17 vs. 8, *p* = 0.009), peripheral enhancement (13 vs. 7, *p* = 0.079), and progressive enhancement curve type (20 vs. 11, *p* = 0.015) were associated with pCR.

**TABLE 3:** DCE‐MRI features before and after NAC according to pathological response

DCE‐MRI features	Before NAC			After NAC		
	pCR (*n* = 24)	non‐pCR (*n* = 24)	*p* value	pCR (*n* = 24)	non‐pCR (*n* = 24)	*p* value
Maximum diameter (mm)	28.9 ± 10.3	31.8 ± 14.4	0.177	11.3 ± 4.3	15.0 ± 9.1	0.005
Reduction in diameter (%)	–	–	–	58.7 ± 16.7	51.1 ± 21.0	0.013
Internal homogeneity			1.000			0.188
Homogeneous enhancement	2(8.3%)	1(4.2%)		5(20.8%)	1(4.2%)	
Heterogeneous enhancement	22(91.7%)	23(95.8%)		19(79.2%)	23(95.8%)	
Enhancement degree			1.000			0.009
Hyper‐enhancement	18(75.0%)	18(75.0%)		7(29.2%)	16(66.7%)	
Iso‐enhancement/hypo‐enhancement	6(25.0%)	6(25.0%)		17(70.8%)	8(33.3%)	
Enhancement order			0.042			0.079
Central enhancement	10(41.7%)	17(70.8%)		11(45.8%)	17(70.8%)	
Peripheral enhancement	14(58.3%)	7(29.2%)		13(54.2%)	7(29.2%)	
Enhancement margin			0.609			1.000
Clear margin	1(4.2%)	3(12.5%)		4(41.7%)	3(12.5%)	
Blurred margin	23(95.8%)	21(87.5%)		20(58.3%)	21(87.5%)	
Enhancement shape			0.348			1.000
Regular shape	1(4.2%)	4(16.7%)		4(41.7%)	3(12.5%)	
Irregular shape	23(95.8%)	20(83.3%)		20(58.3%)	21(87.5%)	
Lymphadenopathy			0.505			0.505
Positive	17(70.8%)	19(79.2%)		17(70.8%)	19(79.2%)	
Negative	7(29.2%)	5(20.8%)		7(29.2%)	5(20.8%)	
DCE curve types			0.763			0.015
Progressive enhancement	9(37.5%)	8(33.3%)		20(58.3%)	11(45.8%)	
Plateau/Washout	15(62.5%)	16(66.7%)		4(41.7%)	13(54.2%)	

Data are expressed as the mean ± standard deviation or *n* (%), and *p* < 0.1 was considered statistically significant.

Multivariate analysis (Table 4) results were as follows: for CEUS features enhancement order before NAC (OR, 8.801; *p* = 0.009), reduction in diameter (OR, 1.031; *p* = 0.094), and enhancement shape after NAC (OR, 18.206; *p* = 0.005) were independently associated with pCR. For DCE‐MRI features, enhancement order before NAC (OR, 7.572; *p* = 0.021), maximum diameter (OR, 0.812; *p* = 0.009), and DCE curve types after NAC (OR, 4.683; *p* = 0.069) were independently associated with pCR.

**TABLE 4:** Multivariate analysis of CEUS features and DCE‐MRI features before and after NAC according to pathological response

CEUS features	Univariate		Multivariate	
	OR (95% CI)	*p* value	OR (95% CI)	*p* value
Enhancement order before NAC	0.294 (0.089–0.974)	0.045	8.801 (1.722–44.988)	0.009
Maximum diameter (mm)	0.882 (0.788–0.988)	0.030	–	–
Reduction in diameter (%)	1.033 (1.004–1.064)	0.028	1.031 (0.995–1.069)	0.094
Internal homogeneity	13.800 (1.582–120.378)	0.018	–	–
Enhancement degree	0.152 (0.029–0.802)	0.026	–	–
Enhancement order	0.388 (0.294–0.974)	0.045	–	–
Enhancement margin	5.000 (1.165–21.459)	0.030	–	–
Enhancement shape	9.308 (1.778–48.723)	0.008	18.206 (2.383–139.095)	0.005
DCE‐MRI features	Univariate		Multivariate	
	OR (95% CI)	*p* value	OR (95% CI)	*p* value
Enhancement order before NAC	0.294 (0.089–0.974)	0.045	7.572 (1.365–41.995)	0.021
Maximum diameter (mm)	0.843 (0.748–0.950)	0.005	0.812 (0.693–0.950)	0.009
Reduction in diameter (%)	1.038 (1.006–1.072)	0.019	–	–
Enhancement degree	0.206 (0.061–0.699)	0.011	–	–
Enhancement order	0.282 (0.083–0.959)	0.043	–	–
DCE curve types	5.909 (1.546–22.580)	0.009	4.683 (0.888–24.688)	0.069

Abbreviations: CI, confidence interval; OR, odds ratio.

### 3.3 Performance of CEUS and DCE‐MRI in predicting pCR

**FIGURE 4:**
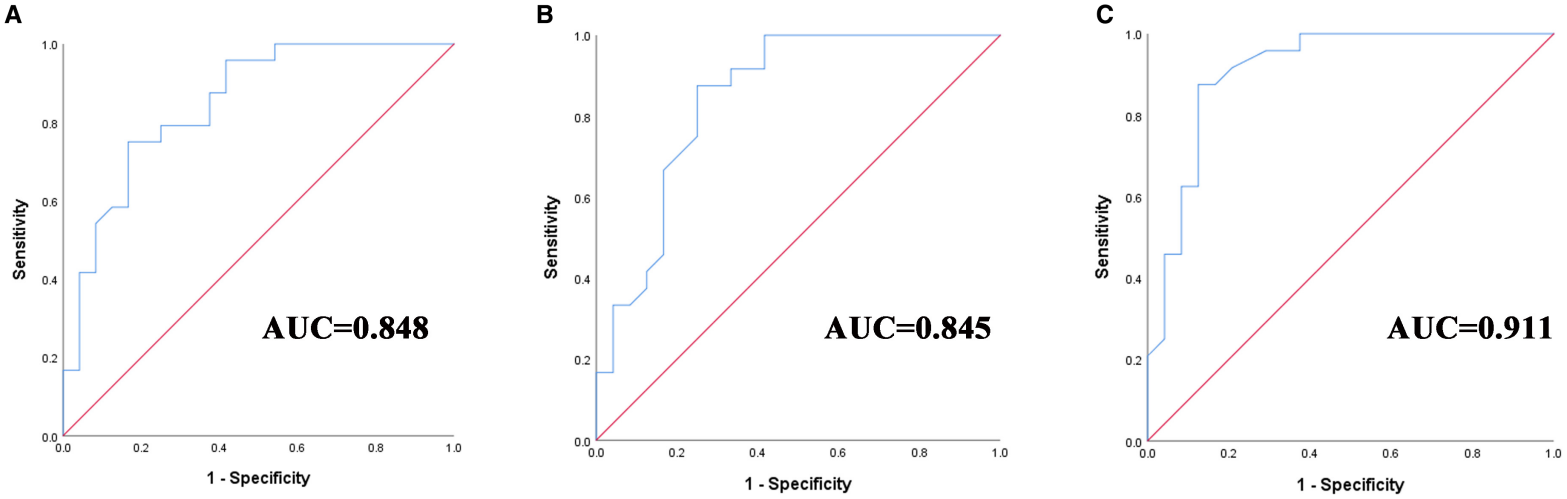
ROC analysis for predicting pCR after NAC in patients with breast cancer. (A) ROC for CEUS. (B) ROC for DCE‐MRI. (C) ROC for CEUS+DCE‐MRI. ROC, receiver operating characteristic; AUC, area under the curve.

ROC analysis was performed to analyze the diagnostic ability of imaging features that were independently associated with pCR after NAC (Figure 4); the results are shown in Table 5. The areas under the ROC curves for CEUS and DCE‐MRI for predicting pCR were 0.848 and 0.845, respectively. The combination of CEUS and DCE‐MRI showed the highest AUC value of 0.911 (95% CI, 0.826–0.997) and significantly higher sensitivity, specificity, accuracy, and PPV for predicting pCR.

**TABLE 5:** Results of the logistic regression analysis

	AUC	Sensitivity	Specificity	Accuracy	PPV	NPV
CEUS	0.848	0.731	0.773	0.750	0.792	0.708
DCE‐MRI	0.845	0.750	0.850	0.792	0.875	0.708
CEUS + DCE‐MRI	0.911	0.840	0.870	0.854	0.875	0.833

Abbreviations: AUC, area under the receiver operating characteristic curve; PPV, positive predictive value; NPV, negative predictive value.

## 4 DISCUSSION

Surgery after NAC has been widely accepted as a critical part of comprehensive treatment for locally advanced breast cancer.^19^ A pathological complete response after NAC has been confirmed to be related to increased overall survival.^20^ However, NAC has been reported to be less effective in 10–35% of patients, triggering breast cancer progression and metastasis.^21^ Thus, effective evaluation of the tumor response after NAC is needed to enable avoidance of ineffective treatment and guide personalized treatment strategies. For patients with an imaging diagnosis of pCR, doctors will be more confident in performing breast‐conserving surgery instead of mastectomy and reducing unnecessary chemotherapy. In contrast, for patients with an imaging diagnosis of non‐pCR, doctors will strengthen the chemotherapy regimen and prolong the chemotherapy cycle to facilitate pCR as much as possible.

Angiogenesis is an important physiological response closely related to tumor growth, invasion, and metastasis.^22^ As chemotherapy induces a decrease in the concentrations of vascular endothelial growth factor and apoptosis of endothelial cells,^23^ altered angiogenesis can be used to detect tumor response after treatment. Previous studies have indicated that quantitative indicators of breast cancer provided by CEUS, such as time to peak and peak enhancement, have value in predicting pathological remission.^13^ However, our analysis of quantitative parameters, such as peak intensity, time to peak, and mean transit time, in this study did not show an obvious association. This may be due to the heterogeneity of breast cancer and differences in computer software analyses. Moreover, qualitative characteristics of CEUS and DCE‐MRI were identified to assess tumor response after NAC in the present study.

Jia et al. demonstrated that CEUS provides useful information regarding tumor blood perfusion changes and distribution features.^24^, ^25^ The capability of CEUS to accurately estimate tumor response after NAC in patients with breast cancer has been previously reported.^26^, ^27^ In the present study, CEUS predicted the pathological response after NAC with AUC value of 0.848, demonstrating significant potential as a detection method for pCR. As chemotherapy can reduce the microvessel density of breast tumors,^28^ the enhancement degree of CEUS also decreased after NAC. Moreover, the irregular shape of the enhanced tumor have been reported to be associated with no pCR.^29^, ^30^

DCE‐MRI is of great value in detecting the response of patients with breast cancer and can accurately evaluate the chemotherapeutic effects.^31^ Tahmassebi et al. reported that DCE‐MRI enabled early prediction of pCR after NAC and the final outcomes of these patients.^32^ As the anti‐angiogenic effect of chemotherapy leads to cell degeneration and apoptosis, which leads to tumor shrinkage,^33^ the smaller maximum diameter detected by DCE‐MRI was independently associated with pCR. Furthermore, Sohrab et al. found that plateau and washout DCE curve types were associated with malignancy.^34^ Similarly, the proportion of plateau/washout DCE curve types in the non‐pCR group was significantly higher.^35^, ^36^

According to our results, both CEUS and DCE‐MRI have considerable potential for assessing the pathological response after NAC in patients with breast cancer, and the diagnostic performance of CEUS seems to be as good as that of DCE‐MRI. Considering its clinical advantages over DCE‐MRI, such as intravascular agent use, low cost, and no contraindications, CEUS seems to be an alternative to DCE‐MRI in some situations. As for the prediction of tumor response to NAC, the combination of CEUS and DCE‐MRI has the highest diagnostic value, with an AUC of 0.911 and a specificity of 87.0%. The combination provided dynamic monitoring of both tumor morphology and perfusion, resulting in a diagnostic performance higher than that of each of the imaging modalities implemented individually. Furthermore, the accuracy and PPV of the combination of CEUS and DCE‐MRI were 85.4% and 87.5%, respectively, which indicate its usefulness for comprehensive evaluation and guiding personalized therapeutic strategies for patients with breast cancer.

Our study has some limitations. This retrospective study was conducted at a single institution, and the study sample was relatively small. Further research is needed on a larger group from multiple institutions to confirm our findings.

## 5 CONCLUSIONS

Our results indicated that CEUS and DCE‐MRI have considerable potential in evaluating the pathological response of patients with breast cancer to NAC, and their combination demonstrated the best diagnostic performance. The study provided a new research direction for predicting the clinical efficacy of NAC.

## AUTHOR’S CONTRIBUTION

Xue Han and Huajing Yang carried out conceptualization. Huajing Yang and Shiyang Jin carried out data curation. Yunfeng Sun carried out formal analysis. Hongxia Zhang carried out investigation. Xue Han and Wen Cheng carried out methodology. Ming Shan carried out project administration. Huajing Yang was involved in writing—original draft. Xue Han was involved in writing—reviewing and editing: Xue Han. All authors gave final approval of the version to be published, and agree to be accountable for all aspects of the work.

## FUNDING INFORMATION

This research did not receive any specific grant from funding agencies in the public, commercial, or not‐for‐profit sectors. All authors declare that they have no conflict of interest.

## CONFLICTS OF INTEREST

All authors declare that they have no conflict of interest.

## ETHICAL STATEMENT

All procedures performed in this study involving human participants were in accordance with the ethical standards of the institutional and national research committee and the 1964 Helsinki declaration and its later amendments or comparable ethical standards. This retrospective study was approved by the ethics committee of our institution, and informed consent was obtained from all patients.

## Data Availability

The datasets generated and analyzed during the current study are available from the corresponding author upon reasonable request. The codes are available from the corresponding author on reasonable request.
